# Hierarchical Dual-Model Detection Framework for Spotted Seals Using Deep Learning on UAVs

**DOI:** 10.3390/ani15213100

**Published:** 2025-10-25

**Authors:** Jun Liu, Fengxiang Jin, Min Ji, Liang Qu, Juan Wang, Chen Wang

**Affiliations:** 1 College of Geodesy and Geomatics, Shandong University of Science and Technology, Qingdao 266590, China; 202383020110@sdust.edu.cn (J.L.); fxjin@sdjzu.edu.cn (F.J.); wangchen20010523@126.com (C.W.); 2 Shandong Engineering Research Center for Beidou Navigation and Intelligent Spatial Information Technology Application, Qingdao 266590, China; 3 Qingdao Key Laboratory of Beidou Navigation and Intelligent Spatial Information Technology Application, Qingdao 266590, China; 4 North China Sea Ecological Center of the Ministry of Natural Resources, Qingdao 266033, China; qingdaoquliang@aliyun.com (L.Q.); 18669718997@163.com (J.W.)

**Keywords:** *Phoca largha*, dual-model architecture, hierarchical object detection, small-object recognition, Unmanned Aerial Vehicle

## Abstract

This study introduces a hierarchical dual-model detection framework for accurately monitoring spotted seals (*Phoca largha*) in the Liaohe River estuary by using deep learning on Unmanned Aerial Vehicles (UAVs). To address challenges such as weak target features, background interference and limited edge computing capacity, this study deploys an optimized FF-YOLOv10 lightweight model on UAVs for rapid target localization, followed by an enhanced PP-YOLOv7 model on ground stations for precise detection. The FF-YOLOv10 model reduces computational complexity by 24.2% and increases inference speed by 33.3%, while the PP-YOLOv7 model achieves 94.2% precision with a 1.9% increase in recall rate. This framework provides an efficient and precise technical solution for the long-term ecological monitoring of marine endangered species, supporting habitat conservation policy formulation and ecosystem health assessments.

## Simple Summary

This study addresses the challenges encountered by Unmanned Aerial Vehicles in monitoring the spotted seal population in the Liaohe River estuary, including the diminishing visibility of small targets, significant background interference and limited edge computing resources. To address these issues, a dual-model layered detection framework is proposed. The framework involves deploying a lightweight object detection model on an Unmanned Aerial Vehicle for the initial screening of spotted seals, followed by precise detection of images transmitted back from the Unmanned Aerial Vehicle using a more accurate detection model on a ground-based workstation. Experimental results demonstrate that this approach not only enhances image processing speed but also significantly improves detection accuracy, reducing both false positives and false negatives. This research study contributes to more accurate population size estimations and dynamic distribution assessments, offering an efficient and reliable method for monitoring endangered species.

## 1. Introduction

Population size is a critical determinant of long-term survival of a species in natural ecosystems and serves as a key indicator of regional biodiversity [1]. The spotted seal (*Phoca largha*), recognized as a keystone indicator of ecosystem health, has gained significant scientific attention due to extensive spatial overlap with human activities across aquatic and terrestrial domains [2,3]. As apex predators, seal populations exert cascading effects on ecosystem dynamics [4]. For instance, in the North Atlantic, robust seal populations may mitigate interspecific competition with commercially vital fish species like flounders, thereby influencing the equilibrium of ecologically and economically significant fish stocks [5]. The Liaohe River estuary, a critical habitat for seals in China, has recently faced growing threats from environmental changes and human activities [6]. Accurately estimating the population size of spotted seals is essential to formulating effective conservation and management strategies to safeguard their habitat and maintain ecosystem stability [7]. In animal population assessments, weak target features, background interference and the constraints of edge computing capabilities significantly influence target recognition accuracy. Studies have indicated that weak target features lead to decreased detection accuracy and increased missed detection rates; background complexity makes target recognition more difficult, and limited edge computing capabilities may lead to real-time response delays, thereby affecting the accuracy and timeliness of monitoring tasks [8,9]. Consequently, the development of efficient and sustainable intelligent monitoring techniques has become a central focus in research on spotted seal monitoring.

Traditional wildlife monitoring methodologies have predominantly relied on invasive techniques, primarily including manual field surveys and GPS collar technology [10], which rely on a large amount of domain knowledge and experience and have insufficient ability to process complex data patterns and high-dimensional data [11]. While these approaches played a pivotal role in early ecological studies, they exhibit inherent limitations, such as high labor intensity, restricted spatiotemporal coverage and substantial invasiveness [12], thereby failing to meet the requirements for large-scale, high-frequency monitoring operations. Unmanned Aerial Vehicle (UAV) technology, characterized by its flexible deployment, high-resolution imaging and multimodal data collection capabilities [13], offers innovative tools for wildlife monitoring [14,15]. Hodgson et al. [16] deployed UAVs equipped with DSLR cameras across varying altitudes, acquiring 6243 high-resolution images that manually identified dugongs with a 95% sighting rate, alongside cetaceans and marine turtles. Kiszka et al. [17] demonstrated UAVs’ utility in shallow coral reef ecosystems by estimating densities of reef-associated elasmobranchs, thereby validating their capacity to deliver critical fishery-independent data in photic zone habitats. In avian ecology, Hodgson et al. [18] also applied drones to estimate the number of bird nests, improving data quality and counting accuracy while showcasing their utility in surveying populations and locations that are otherwise difficult to access. Beaver et al. [19] further advanced this paradigm by integrating thermal infrared sensors on UAVs to survey white-tailed deer populations, where independent observers achieved significantly higher detection probabilities compared with manned aerial surveys. Notwithstanding the demonstrated reliability of manual animal identification in UAV imagery, the exponentially growing volume of image datasets necessitates labor-intensive interpretation, resulting in suboptimal efficiency and operator-dependent biases [20]. Consequently, the development of intelligent wildlife auto-recognition algorithms has become essential to overcoming the technical challenges in monitoring [21,22].

Deep learning is a machine learning technique based on artificial neural networks. Its core principle lies in progressive feature extraction and nonlinear transformation, which enables multi-level feature learning and automatic extraction of high-level semantic features from data [23]. In recent years, with the advancement of artificial intelligence technologies, deep learning-based object detection models have provided valuable technical support for wildlife monitoring. Peng et al. [24] improved the Faster R-CNN model by optimizing anchor scales and addressing difficult negative samples, resulting in an increase in the F1 score for detecting Tibetan wild donkeys from 44% to 86%, significantly reducing the need for manual labor. Gray et al. [25] applied a convolutional neural network (CNN) to detect sea turtles in drone images, finding that the model detected 8% more turtles compared with manual counting, greatly enhancing detection accuracy. Tripathi et al. [26] employed single-stage detection models, including YOLO and DETR networks, for non-invasive, real-time detection of swamp deer to estimate their population size. Jiang et al. [27] proposed an enhanced wilDT-YOLOv8n, which integrates deformable convolution and multimodal attention mechanisms, boosting the mean average precision for wildlife detection to 88.54% and achieving a tracking accuracy of 40.35%, thus reducing target loss caused by obstacles. Wu et al. [28] utilized the improved InceptionResNetV2 model, incorporating dual attention mechanisms and Dropout optimization, to achieve 99.37% identification accuracy for individual Amur tiger stripes, providing algorithmic support for the precise conservation of endangered species.

Although deep learning and drone technology hold significant promise for the future of wildlife monitoring—with UAV-based counts of colony-nesting birds showing greater precision than traditional ground counts [18]—they still face ecological and technical limitations. Lightweight models are essential to meeting the endurance and computational constraints of UAVs; for example, the YOLOv8-E model incorporates edge-sensitive Sobel-based modules to significantly reduce computational cost while maintaining detection accuracy [29]. Similarly, LPS-YOLO has demonstrated improved detection accuracy for small targets in UAV imagery despite parameter reduction [30]. However, animal detection from drone images remains challenging due to small target size, complex backgrounds and the limited distinguishable features inherent in such data [31]. Moreover, balancing wide-area coverage with precise species identification remains unresolved. The MDTS framework addresses this by combining thermal detection for broad coverage with high-resolution RGB zoom for accurate identification, significantly reducing data volume while enhancing ecological survey efficiency [32]. On the other hand, systems like WildLive achieve near real-time detection and tracking onboard UAVs—processing HD and 4K video at frame rates of 17 FPS and 7 FPS or greater, respectively, demonstrating feasibility but also highlighting the resource limits even for advanced onboard hardware [33]. To address these challenges, we propose a dual-model hierarchical framework that integrates lightweight UAV-based detection with high-precision ground-station verification. Unlike existing approaches that emphasize either lightweight efficiency or high-accuracy detection alone, our method synergistically bridges both, achieving real-time aerial monitoring with precise post-verification. This framework offers a practical, energy-efficient solution for small marine mammal ecological monitoring, enhancing drone operational endurance and complementing the current literature on UAV-aided conservation.

In summary, UAV and deep learning technologies provide promising tools for wildlife monitoring but face persistent challenges, such as weak target features, background interference and limited onboard computing resources. To address these issues, this study proposes a dual-model hierarchical detection framework that combines UAV-based lightweight detection with high-precision ground-station verification, thereby balancing efficiency and accuracy in spotted seal monitoring. The main contributions of this study are as follows:

(1) A dual-model hierarchical detection framework is developed, integrating UAV-based lightweight detection with high-precision ground-station verification to achieve the real-time monitoring and accurate population estimation of spotted seals.
(2) A lightweight YOLOv10 [34] variant optimized for edge deployment is constructed, incorporating focal modulation networks (FocalNets) to enhance the detection of hard-to-recognize targets under limited onboard resources.
(3) The ground-based YOLOv7 [35] model is enhanced with multi-scale feature pyramids and partial convolution, strengthening small-target representation and suppressing background interference, thereby achieving a practical balance between accuracy and efficiency.

## 2. Materials and Methods

### 2.1. Study Area

**Figure 1:**
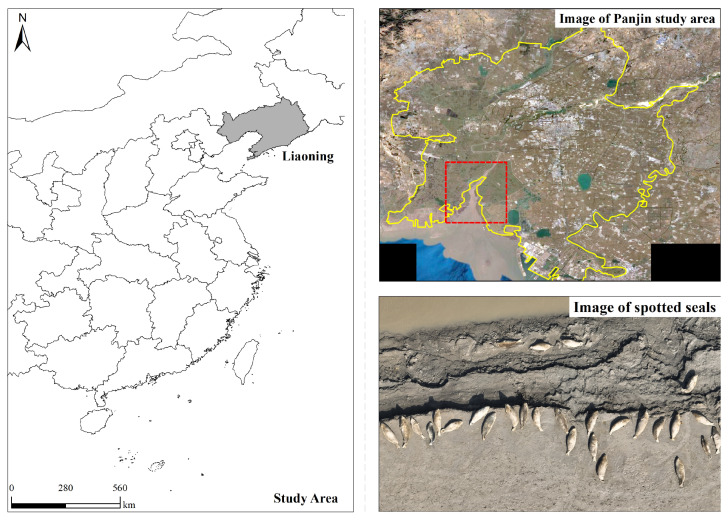
Overview of the study area. The area within the red rectangle represents the region captured by the drone imagery.

Liaohekou, as shown in Figure 1, the southernmost breeding area for the harbor seal in the western Pacific, is home to a coastal wetland ecosystem of considerable ecological importance [36]. This region is characterized by distinctive habitat conditions, including brackish salt marshes and mudflats resulting from the confluence of seawater and freshwater, along with seasonal sea ice coverage during the winter months. These unique environmental features support a food chain for the harbor seal, primarily consisting of benthic organisms and fish. This unique ecotone features characteristic habitats, including saline marshes and mudflats formed by brackish water convergence, coupled with seasonal ice cover during winter [6]. The region is situated at the junction of Bohai Bay and the Liaohe Alluvial Plain, functioning as an essential stopover on the East Asia–Australasia migratory route for migratory birds. It is also the sole breeding habitat of the spotted seal in Chinese waters, offering significant ecological connectivity and conservation value.

### 2.2. Data Acquisition

**Figure 2:**
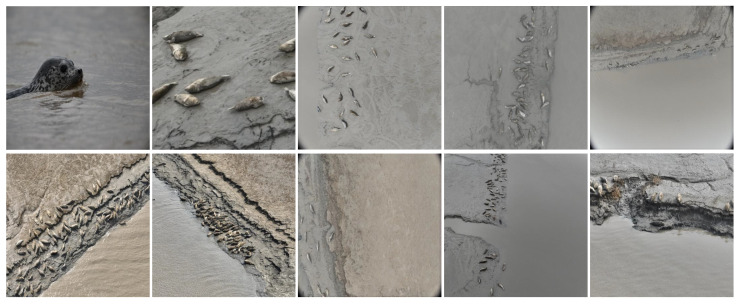
Raw images used for annotation and augmentation.

To obtain images of seals in their natural habitat, photographs were sourced from various natural environments, and the DJI Mavic 3E drone (Shenzhen, China) was utilized to capture images at the Shuangtaizi estuary, with flight altitudes set at 15 m and 20 m. Given the challenges in acquiring spotted seal image data and the relatively limited number of individuals inhabiting coastal areas, this study employed data augmentation techniques—including spatial geometric transformations, edge padding and complex background scaling—to expand the collected spotted seal imagery. These methods were applied to increase both the quantity and diversity of training samples, thereby improving model performance during training and enhancing the generalization capability of the computational framework. Typical collected images are shown in Figure 2, which illustrate the raw dataset diversity and field conditions prior to annotation. These examples provide an overview of the visual complexity encountered in natural habitats and highlight the challenges of data acquisition.

**Figure 3:**
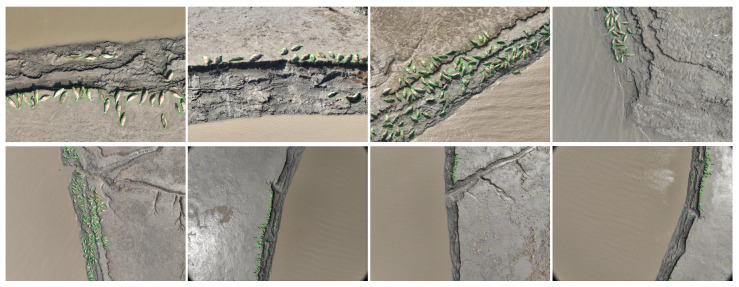
Labeled images with bounding boxes around spotted seals for model training.

The augmented images were annotated using the Labelimg tool, with the minimum enclosing rectangles drawn around the seals, ensuring minimal background inclusion within the rectangle. The class attribute for each rectangle was designated as “*Phoca largha*”. After completing the annotations, an XML format label file was generated, containing the height, width and class information for each rectangle. The annotated images are shown in Figure 3, serving as examples of the labeling strategy applied in this study. These figures demonstrate how bounding boxes were drawn to capture the seals while minimizing background noise, thereby establishing the ground-truth labels used for subsequent model training and evaluation.

### 2.3. Real-Time Spotted Seal Detection Model Based on Enhanced YOLOv10

**Figure 4:**
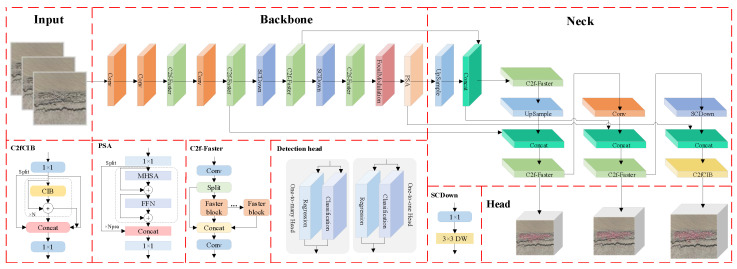
Structure of FF-YOLOv10.

YOLOv10 is a real-time, end-to-end object detection model based on the YOLOv8 architecture [37]. It employs a consistent dual-assignment strategy, incorporating dual-label assignment and consistency matching metrics, eliminating the need for NMS and effectively addressing redundant predictions during post-processing. Based on this model, this paper proposes the FF-YOLOv10 model for real-time detection of spotted seals by drones. The structure of this model is shown in Figure 4. The FF-YOLOv10 model incorporates FasterNet [38] to streamline the C2f module, optimizing the network structure and reducing the number of parameters, thereby meeting the requirements for efficient deployment on mobile devices. Given the complex habitat of seals, some targets are challenging to detect due to issues such as positional overlap and image blurriness. To address these challenges, the SPFF module is replaced by FocalNets, which improves the model’s ability to focus on hard-to-detect targets by adjusting feature map responses, thus enhancing overall detection performance.

#### 2.3.1. Lightweight C2f Module

The FasterNet module, known for its efficiency, is widely adopted in object detection algorithms due to its exceptional speed and effectiveness across various visual tasks. This architecture improves feature representation and expands the receptive field while maintaining a lightweight design and high processing speed. Additionally, it emphasizes the importance of simplifying the computation process by minimizing redundant elements. This approach not only optimizes computational resource utilization but also significantly enhances the network’s processing efficiency.

**Figure 5:**
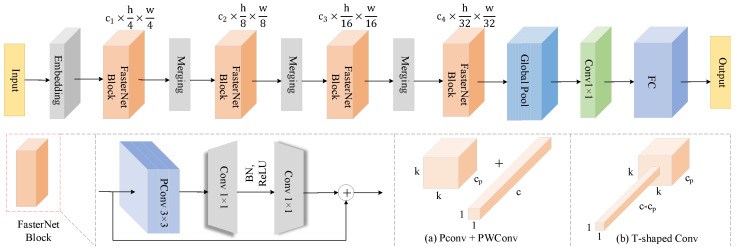
Overall architecture of FasterNet. The top shows the network pipeline, while the bottom illustrates the structure of the FasterNet Block and two convolution strategies.

The FasterNet architecture, as illustrated in Figure 5, adopts a modular design to ensure both flexibility and scalability. It begins with PConv, which selectively applies spatial convolutions to specific input channels, reducing redundant computation and floating-point operations while preserving key spatial features. This mitigates the accuracy loss often associated with deep convolutions. Subsequently, PWConv enables channel expansion and local cross-channel interaction, forming a T-shaped structure whose weight distribution aligns with pre-trained network statistics, enhancing central receptive field focus. FasterNet adopts a four-stage hierarchical structure in which the shallow stage utilizes high-resolution features and dense PConv modules to extract fine-grained details, while the deeper stages progressively increase channel dimensions to enhance semantic abstraction. Spatial downsampling and channel expansion are simultaneously optimized through embedding or merging layers. Normalization and activation are introduced only after the intermediate PWConv, and residual connections are employed to maintain feature diversity and gradient stability, thereby avoiding information loss caused by excessive normalization. For efficient hardware deployment, the architecture minimizes memory access conflicts by simplifying branches and reduces data transfer via cross-channel feature reuse. Serialized variants offer a tunable balance between accuracy and speed. The overall design ensures low latency, high accuracy, and cross-platform adaptability, making it effective for visual tasks in resource-constrained environments.

To address the challenge of large model sizes impacting detection speed and hindering drone deployment in seal detection tasks, this paper incorporates the fast convolutional structure of FasterNet into the C2f module, as illustrated in Figure 4. Upon inputting a feature map with dimensions h (height), w (width) and c (channel) into the C2f-Faster module, the input feature map is first processed by convolution and then split into two parts: one is passed through directly, while the other undergoes additional processing through multiple FasterNet modules. The concatenated feature map is subsequently processed by another convolutional layer to generate the final output feature map [39]. In the FasterNet module, the feature map is partitioned into several subregions, with some undergoing convolution operations, which are then combined with the unprocessed subregions. This design enables the seal detection model to operate efficiently on drones with limited memory, facilitating rapid detection while reducing model complexity and significantly improving the speed of object detection tasks.

#### 2.3.2. Focal Modulation Networks

**Figure 6:**
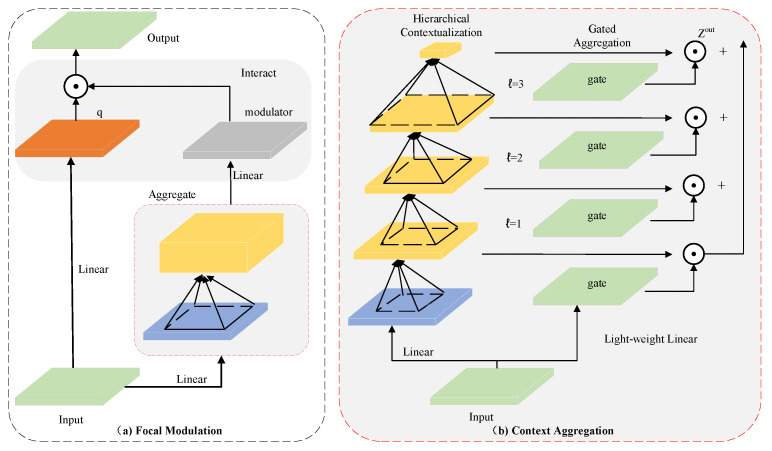
Focal modulation network architecture.

To effectively integrate contextual information at different granularities for visual representation learning, this study adopted the Focal Modulation Networks architecture, as illustrated in Figure 6. Moreover, by selectively emphasizing relevant features, FocalNets effectively mitigates background noise, ensuring precise recognition and localization of spotted seals. The implementation of the algorithm is detailed below.

(1) Focal Modulation

Given an input visual feature map $X\in R^{C\times H\times W}$, a linear projection is first applied to obtain a set of query features *q*. Meanwhile, contextual information is aggregated using a contextual encoder $M_{2}$, and a lightweight interaction function $T_{2}$ is applied to modulate the query with the contextual signal. The output representation at each spatial location *i* is computed as

(1) $$\begin{array}{c} y_{i}=T_{2}\left(M_{2}\left(i,X\right),x_{i}\right) \end{array}$$

Here, $M_{2}$ captures multi-scale contextual cues from the surrounding neighborhood, and $T_{2}$ denotes an element-wise interaction operator, such as addition or modulation, that fuses the context with the query in a content-adaptive manner.

(2) Hierarchical Contextualization

To capture semantic context at multiple scales, we adopt a hierarchical representation strategy. First the input *X* is projected onto a new feature space via a linear transformation:

(2) $$\begin{array}{c} Z^{0}=f_{z}\left(X\right)\in R^{H\times W\times C} \end{array}$$

Subsequently, we apply *L* layers of depth-wise convolutions (DWConv) with GelU activations to progressively expand the receptive field and encode increasingly global context:

(3) $$\begin{array}{c} Z^{\left(\ell \right)}=\mathrm{GeLU}\left(\mathrm{DWConv}\left(Z^{\left(\ell -1\right)}\right)\right),\;\ell =1,\ldots ,L \end{array}$$

Each layer $Z^{\ell }$ captures context at a specific spatial scale. To include global semantics, we apply average pooling to the final output:

(4) $$\begin{array}{c} Z^{\left(L+1\right)}=AvgPool\left(Z^{\left(L\right)}\right) \end{array}$$

This results in L+1 contextual feature maps, which together span a continuum from fine-grained local to coarse global representations.

(3) Gated Aggregation

A gated aggregation mechanism is introduced to adaptively fuse multi-scale contextual features. Specifically, a linear layer is employed to obtain the spatial and hierarchical awareness weights $G=f_{g}\left(X\right)\in R^{H\times W\times \left(L+1\right)}$. A weighted sum is then computed via element-wise multiplication to produce a single feature map $Z^{out}$, maintaining the same spatial dimensions as the input *X*:

(5) $$\begin{array}{c} Z^{out}=\sum_{\ell =1}^{L+1}G^{\ell }⊙Z^{\ell }\in R^{H\times W\times C} \end{array}$$

where $G^{\ell }\in R^{H\times W\times C}$ denotes a slice of *G*, corresponding to the ℓ−th focus layer on the horizontal plane. This design allows FocalNets to adaptively learn and integrate information from different focal depths. To facilitate information propagation across different channels, another linear layer is applied to generate the focused control mapping $M=h\left(Z^{out}\right)\in R^{H\times W\times C}$. The final focused control operation is formulated as

(6) $$\begin{array}{c} y_{i}=q\left(\alpha \left(x_{i}\right)\right)⊙\left(\sum_{\ell =1}^{L+1}g_{i}^{\ell }\cdot z_{i}^{\ell }\right) \end{array}$$

where $g_{i}^{\ell }$ and $z_{i}^{\ell }$ represent the control value and visual features at the *i*-th spatial location in $G^{\ell }$ and $Z^{\ell }$, respectively.

### 2.4. Improved YOLOv7-Based Precision Detection Model for Spotted Seals

**Figure 7:**
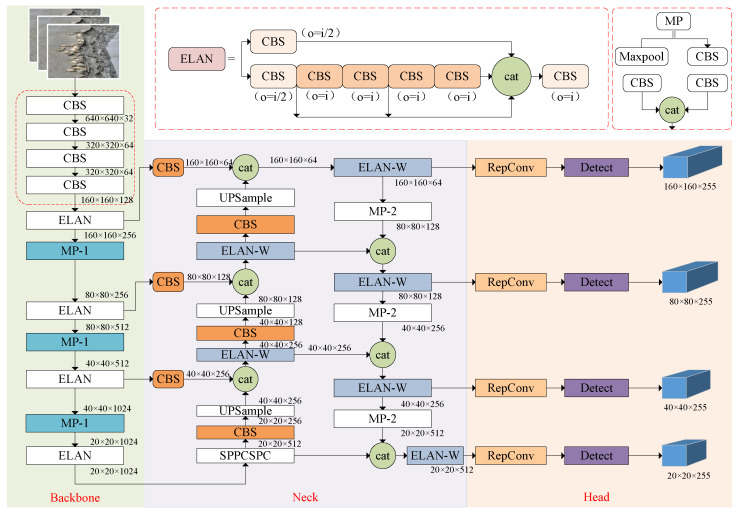
Structure of PP-YOLOv7.

The YOLOv7 model is primarily composed of the backbone network, the neck network and the detection layer. The backbone network utilizes the ELAN structure for feature extraction, enhancing feature representation capabilities while maintaining consistent input and output feature sizes. Additionally, max pooling (MP) is applied for downsampling, using both max-pooling and convolution operations while adjusting the number of channels. The neck network integrates the CBS, SPPCSPC, MP and ELAN modules, adhering to the traditional PAFPN architecture. This network extracts multi-scale features from the backbone for comprehensive feature fusion. Finally, the REPConv architecture is employed to design reparameterized convolutions, balancing network complexity during training while reducing parameters and computational cost during inference, without compromising accuracy. To mitigate false positives and missed detections of small targets in UAV images caused by low resolution and sparse feature information, an improved YOLOv7 model is deployed at the ground workstation. As illustrated in Figure 7, the PP-YOLOv7 architecture incorporates a small-object detection module, which enhances the representation of small targets by capturing spatial details and contextual information more effectively. In addition, the use of partial convolution focuses computation on the regions of interest within the input image, thereby suppressing interference caused by background regions with similar colors. This design significantly improves the model’s generalization capability and robustness.

#### 2.4.1. Small-Target Detection Layer

With input images of 480 × 480 pixel resolution, the original YOLOv7 network employs three distinct detection scales (20 × 20, 40 × 40 and 80 × 80) for feature map analysis to accommodate multi-scale targets. However, in UAV-based wildlife monitoring scenarios, particularly for protected species like spotted seals, image acquisition requires maintaining sufficient shooting distances to avoid disturbing natural behaviors, inevitably introducing complex backgrounds and multi-scale targets within captured imagery. Small targets, characterized by low pixel occupancy and limited visual features, often suffer from ineffective recognition in such contexts. The original model demonstrates suboptimal performance in small-target detection due to the restricted receptive field of its 80 × 80 scale feature maps, leading to compromised precision due to either false positives or missed detections.

**Figure 8:**
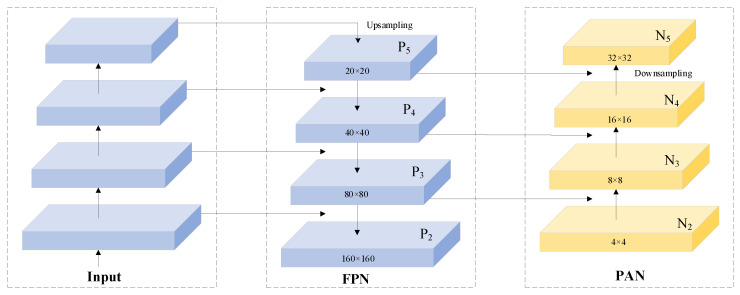
Schematic of small-target detection layer. The input is used for image data input, FPN represents multi-scale feature extraction, and PAN is used for feature enhancement and information flow.

To address this limitation, we introduce a dedicated small-target detection layer that preserves and constructs higher-resolution shallow feature maps for direct participation in detection predictions. These high-spatial-resolution features enhance the model’s representational capacity by capturing subtle textures and contour features of small targets, thereby significantly reducing both missed detection rates and false detection rates. Specifically, our implementation first upsamples the 80 × 80 scale feature maps generated by the FPN module to obtain 160 × 160 resolution representations. Subsequently, these upsampled features are fused with shallow-layer features extracted from the backbone network, forming enhanced 160 × 160 feature maps that are directly fed into the prediction module. This integration strengthens the model’s capability to detect small targets through multi-scale feature preservation. Representative examples of output feature maps across four detection scales are illustrated in Figure 8.

#### 2.4.2. Partial Convolution

**Figure 9:**
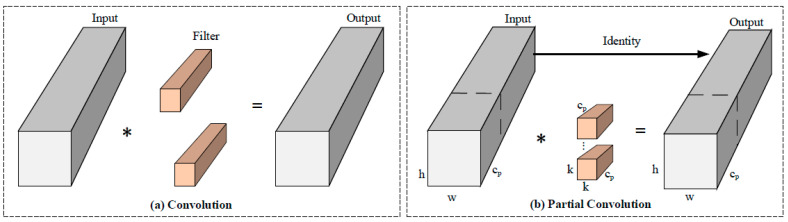
Conv and PConv structures. * denotes the convolution.

In contrast to conventional convolution (Conv) modules, PConv operates exclusively on a specific fraction of input feature map channels rather than simultaneously processing all channels. As shown in Figure 9, this selective processing mechanism applies convolution operations to strategically chosen channel subsets while preserving the integrity of unmodified channels. The processed outputs are subsequently concatenated with bypassed channels through residual connections, maintaining comprehensive feature representation in final outputs. By optimizing both computational channels and memory access operations, this architecture achieves significant reductions in FLOPs and memory usage compared with standard Conv modules while maintaining equivalent model performance metrics.

The high-resolution imagery acquired by UAVs for monitoring Phoca largha generates substantially increased pixel processing demands during analytical workflows. Traditional convolutional neural networks, typically constructed as multi-layered hierarchical architectures, involve successive convolution operations and feature abstraction through depth-wise progression. While this layered configuration effectively captures high-level semantic features, it imposes significant computational burden and memory footprint—particularly evident in the memory access complexity defined by the following operational formula:

(7) $$\begin{array}{c} h\times \omega \times 2c+k^{2}\times c^{2}\approx h\times \omega \times 2c \end{array}$$

PConv minimizes computational redundancy through optimized convolutional operator design, achieving dual reduction in arithmetic complexity and memory consumption. When employing a standard channel selection ratio of $r=c_{p}/c=1/4$, the memory access operations for PConv can be mathematically expressed as

(8) $$\begin{array}{c} h\times \omega \times 2c_{p}+k^{2}\times c_{p}^{2}\approx h\times \omega \times 2c_{p} \end{array}$$

It amounts to only one-fourth of the computational cost of a standard convolution.

In seal detection tasks, the high similarity between the gray-black speckled patterns on the seals’ dorsal regions and the muddy tidal flat backgrounds often introduces background interference, leading to missed detections or false positives; therefore, it is crucial to effectively extract local features within the region of interest and integrate them into global features [40]. To address this, PConv employs masks to focus computational resources on regions of interest within the input image, thereby enabling more precise extraction of seal-related features. This process effectively suppresses background noise, emphasizes the morphological and textural characteristics of spotted seals, and significantly enhances detection accuracy and robustness. Furthermore, spotted seal detection faces challenges arising from variations in individual appearance, posture and behavioral patterns. By leveraging PConv to improve the model’s generalization capability, the network adaptively captures discriminative features across diverse scenarios, ensuring robust performance under variations in seal appearance and behavior. Consequently, replacing the CBS convolutional layer in the second branch of the ELAN structure with the improved PConv architecture yields superior performance. Compared with traditional convolution, PConv demonstrates enhanced proficiency in processing image edges and fine-grained details, which facilitates more accurate boundary localization in subsequent detection tasks.

## 3. Results

### 3.1. Environmental Configuration and Evaluation Metrics

In order to ensure the fairness of the results, the parameter settings of the model in this paper are uniformly set to 300 epochs, a batch size of 32 and an image pixel size of 480 × 480, and other parameters are set to default values. Both the final iteration’s model weights and the optimal weights were saved for subsequent analysis. The augmented dataset comprises 3036 images, which were split into training and validation sets in a 9:1 ratio to ensure no overlap between the two sets, thereby mitigating the risk of model overfitting. The experimental hardware and software environment is detailed in Table 1.

To systematically evaluate the performance improvements of the proposed model, this study adopts the following metrics: precision (P), recall (R), mean average precision (mAP), frames per second (FPS) and model weight file size (MB). The model weight file size (MB) serves as an indicator of model complexity, where a smaller file size corresponds to reduced computational demands. FPS quantifies the real-time capability and efficiency of the object detection algorithm by measuring the number of image frames processed per second. The mathematical formulations for precision, recall and mAP are provided below:

(9) $$\begin{array}{c} Precision=\frac{TP}{TP+FP} \end{array}$$

(10) $$\begin{array}{c} Recall=\frac{TP}{TP+FN} \end{array}$$

(11) $$\begin{array}{c} mAP=\frac{1}{N}\sum_{n\in N}AP\left(n\right) \end{array}$$

In the equations, TP denotes the number of correctly identified positive samples; FP represents the number of negative samples erroneously classified as positive instances, i.e., erroneous detections; FN corresponds to the number of positive samples incorrectly predicted as negative instances, reflecting missed detections; AP(n) is defined as the area under the PR curve for the *n*-th category of detection targets; and *N* indicates the total number of categories in the evaluation.

### 3.2. Selection of Baseline Models for UAVs and Ground Stations

Given the varying recognition efficacy of different algorithms on the spotted seal dataset, this study conducted systematic comparisons between the proposed algorithm and mainstream object detection algorithms to justify the selection rationale of the baseline algorithm and demonstrate the superiority of the improved method. As evidenced by the experimental results in Table 2, YOLOv11 [41] achieves higher FPS than YOLOv10, albeit with marginally reduced precision and recall rates. Compared with the YOLOv8 model, YOLOv10 delivers superior precision and recall with lower computational load, while its reduced memory footprint and high frame rate prove particularly advantageous for video surveillance and mobile device applications. The rapid processing capability enables timely detection and response to dynamic targets in video streams. Among all models, YOLOv7 demonstrates the highest precision and recall rates, making it suitable for scenarios requiring extreme accuracy. However, despite its detection accuracy advantages, YOLOv7 exhibits inferior detection speed compared with YOLOv10. Under resource-constrained conditions, YOLOv10 maintains real-time detection performance with lower computational demands, enabling effective deployment on UAV mobile platforms while preserving practical detection accuracy. This comparison aligns with recent findings, which indicate that YOLOv10 outperforms YOLOv7 in terms of speed, size, and latency, while maintaining comparable accuracy. This makes YOLOv10 particularly advantageous for UAV inference tasks in resource-constrained environments [42]. In contrast, YOLOv7 continues to demonstrate strong baseline precision, especially in complex scenarios such as UAV imaging over water surfaces, which makes it highly suitable for secondary verification tasks in ground-station applications [43,44]. Consequently, for the spotted seal dataset, YOLOv7 was selected as the baseline model for subsequent experiments to meet the ground workstation’s stringent accuracy requirements, whereas YOLOv10 serves as the foundational model for real-time UAV deployment applications.

### 3.3. Comparison and Ablation Experiments of UAV End Models

#### 3.3.1. Comparison Experiment of Backbone Network

**Figure 10:**
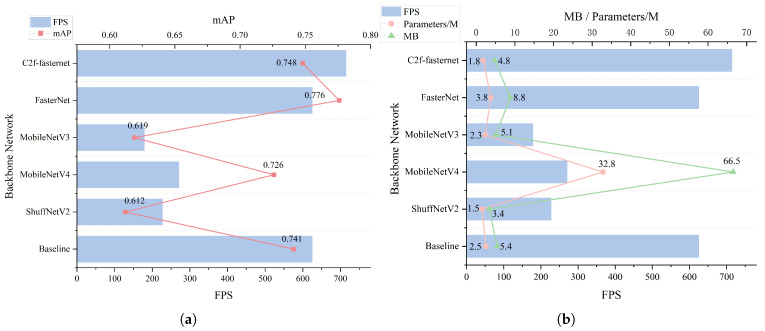
Performance comparison of different networks in YOLOv10. (**a**) FPS-based mAP comparison. (**b**) FPS-based comparison of MB and parameters.

To validate the performance of different lightweight models in YOLOv10, this study selected widely adopted lightweight architectures, including ShuffNetV2 [45], the MobileNet series [46,47] and FasterNet, for a series of comparative experiments. Figure 10 presents evaluation metrics across different networks, comparing the performance impact of the improved C2f-Faster module and existing lightweight networks on the spotted seal dataset. As demonstrated in Figure 10, the proposed method achieves higher inference speed and accuracy compared with networks with fewer parameters. Furthermore, when benchmarked against networks maintaining acceptable accuracy ranges, C2f-Faster effectively reduces parameter counts and computational complexity while sustaining high inference speeds to ensure processing timeliness.

#### 3.3.2. Comparison with Other UAV End Models

To validate the superiority of the proposed FF-YOLOv10 network over conventional object detection algorithms, we trained multiple state-of-the-art models on a custom dataset. For experimental reliability, Spike-YOLO [48], YOLOv7-tiny, RT-DETR [49] and SSD [50] were trained using identical hyperparameters to their original unmodified versions, as shown in Table 3. Unlike traditional accuracy-focused evaluation, our emphasis here is on FPS, MB and parameter count, which are critical to real-time onboard deployment on UAV platforms. Comparative results with other YOLO algorithms from Table 2 demonstrate that the FF-YOLOv10 model outperforms nine competing models in lightweight metrics for spotted seal detection tasks, specifically regarding parameter count and weight file size, although its weight file size remains larger than that of YOLOv10. Additionally, with minimal loss in AP and recall, the proposed model achieves significant detection speed improvements through its optimized network architecture, effectively meeting real-time detection requirements. Alternative methods designed for rapid UAV-based spotted seal detection and data acquisition exhibit limited generalizability, struggling with insufficient detection accuracy and suboptimal inference speeds. Consequently, the selection of FF-YOLOv10 as the onboard detection algorithm for UAV systems demonstrates strong justification. This choice not only ensures high detection accuracy and rapid inference capabilities but also substantially enhances practical applicability and operational effectiveness in field deployments.

#### 3.3.3. Ablation Experiment of UAV End Models

**Figure 11:**
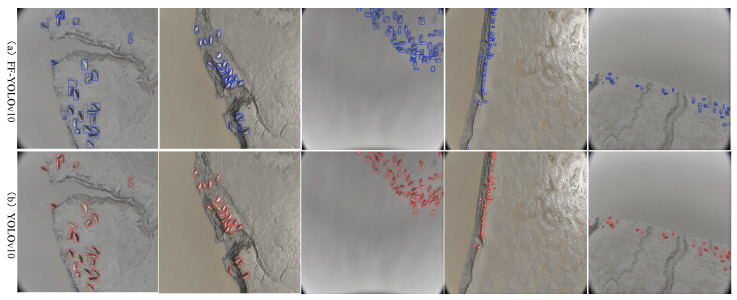
Visualization results of the FF-YOLOv10 model detecting spotted seals in UAV images captured from different altitudes. The model demonstrates robust detection performance across varying scales and resolutions.

To evaluate the individual impact of each improved module on FF-YOLOv10 (a lightweight model designed for UAV deployment), we conducted a series of ablation experiments on the spotted seal dataset. The final ablation results are presented in Table 4. As shown in the table, after replacing the original C2f module, the modified model demonstrates a 28.6% reduction in parameters and an 11.1% decrease in weight file size compared with the baseline YOLOv10. Concurrently, it achieves a 14.3% improvement in inference speed and a 0.9% enhancement in mAP. For single-class detection on the spotted seal dataset, this mAP improvement directly corresponds to a 0.9% increase in seal detection accuracy. These results indicate that while the C2f-Faster module causes a 1.2% reduction in recall rate, it effectively reduces model size, accelerates detection speed and enhances feature extraction capabilities. The final FF-YOLOv10 model, incorporating FocalNets into F-YOLOv10, further improves recall rate and inference speed. Compared with YOLOv10, its inference speed has improved by 33.3%. Meanwhile, the parameter count and weight file size are reduced to 75.8% and 92.6% of the original values, respectively. This demonstrates that UAVs equipped with the optimized model can process imagery more rapidly in real-time operations. Through higher frame rates, such systems capture more critical frames while mitigating false negatives or positives caused by motion blur or fast-moving targets. To intuitively demonstrate the detection capability of the FF-YOLOv10 model onboard UAVs, Figure 11 presents visualization results of spotted seal detection at different flight altitudes. The images were captured at varying UAV heights to reflect typical aerial monitoring scenarios. As shown in the figure, FF-YOLOv10 effectively identifies seals across scales, maintaining reliable localization performance even when targets appear small due to high-altitude imaging. This highlights the model’s suitability for fast onboard inference during large-area search operations.

### 3.4. Comparison and Ablation Experiments of Ground-Station Models

#### 3.4.1. Comparison with Other Ground-Station Models

**Figure 12:**
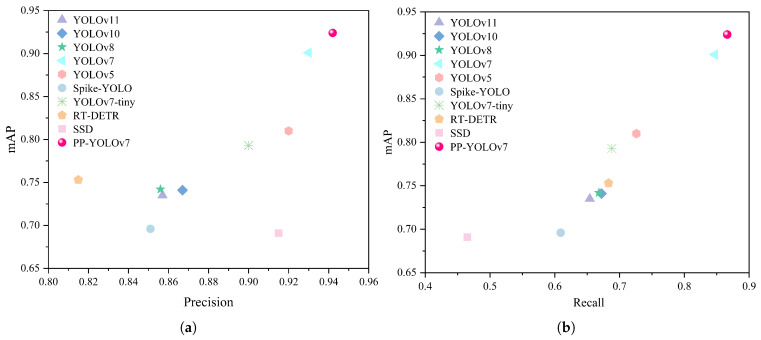
Experimental comparison of classical object detection networks. (**a**) Precision comparison based on mAP. (**b**) Recall comparison based on mAP.

This study conducts a comprehensive performance comparison between the proposed PP-YOLOv7 algorithm and state-of-the-art object detection methods for spotted seal detection tasks. The evaluation metrics primarily include mean average precision, precision and recall. To intuitively visualize the performance disparities across algorithms, a scatter plot is employed for visualization, as illustrated in Figure 12. The improved model achieves 94.2% precision and 86.6% recall, surpassing a series of benchmark algorithms. This demonstrates that PP-YOLOv7 achieves an optimal balance between detection accuracy and robustness. Compared with the original YOLOv7 model, the proposed method improves precision and recall by 1.2% and 1.9%, respectively, in spotted seal detection. For single-class spotted seal detection tasks, the model attains an mAP of 92.4%, highlighting its exceptional detection performance in significantly enhancing the reliability and efficiency of target recognition. These advancements are critical to the large-scale ecological monitoring of spotted seals. The high precision effectively reduces false positives in ground workstation-based monitoring, ensuring that only genuine targets are identified and recorded. This minimizes interference from invalid data and enhances the overall efficiency of the monitoring system. Furthermore, ground-station monitoring tasks typically require extensive spatial coverage, where any missed detection of individual seals could adversely impact population assessments, conservation policy formulation and environmental impact evaluations. The high recall of PP-YOLOv7 guarantees comprehensive detection coverage, thereby providing reliable data support for the long-term monitoring of spotted seal population dynamics.

**Figure 13:**
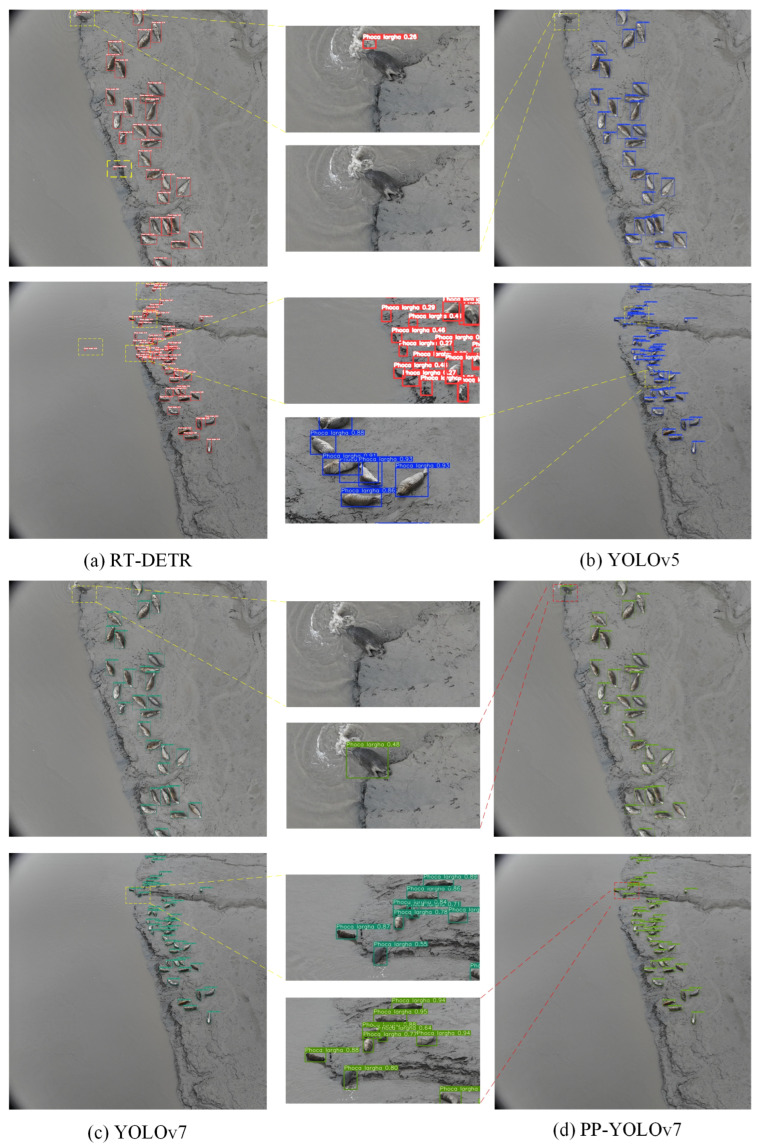
Visualization of model detection results. The yellow dashed line represents the detection challenges faced by other models, while the red dashed line illustrates the notable improvement achieved by PP-YOLOv7. The red, blue and green rectangles in the image correspond to the detection performance of the model.

The above analysis provides an objective evaluation of the model’s improvement effects. To more intuitively demonstrate the superior performance of the enhanced model, selected detection results are visualized in Figure 13. The RT-DETR model fails to extract sufficient semantic information to distinguish background elements, resulting in a detected spotted seal count that far exceeds the actual number and a significantly higher false positive rate compared with other algorithms. Due to variations in the proportion of spotted seals within the frame and resolution limitations, both YOLOv5 and YOLOv7 exhibit varying degrees of missed detections and false positives. In contrast, the proposed PP-YOLOv7 algorithm, equipped with a small-target detection layer, achieves precise identification of spotted seals of varying sizes in complex environments. Additionally, its prediction bounding boxes align more accurately with actual targets. As a ground workstation detection model, PP-YOLOv7 effectively reduces both missed detection rates and false positive rates, demonstrating marked improvements in scenarios involving overlapping seals and background interference.

#### 3.4.2. Ablation Experiment of Ground-Station Models

To objectively evaluate the precision of the improved YOLOv7 model in detecting multi-scale spotted seal targets within complex UAV scenarios using real-time transmitted data, we systematically integrated enhancement modules into the baseline YOLOv7 and assessed their individual impacts through ablation studies, with results detailed in Table 5. The incorporation of a small-target detection layer into the baseline model increased accuracy, recall and mAP by 0.7%, 3.2% and 2.9%, respectively, while reducing model complexity and decreasing weight file size by 3.1%. These results demonstrate that the small-target detection layer significantly enhances holistic detection performance, particularly improving recognition capability for small objects in complex backgrounds. Building upon this, the integration of PConv further elevated detection accuracy and mAP to 0.942 and 0.924, respectively, without increasing parameter count or weight file size. Although the recall rate slightly decreased compared with the model with only the small-target detection layer, it remained 2.2% higher than the baseline. This indicates that the optimized model strengthens detection robustness across diverse environmental conditions. By enabling accurate quantification of seal populations and reliable assessment of their ecological status, the enhanced system ensures dependable monitoring performance.

### 3.5. Comparison of Detection Results Under Different Weather Conditions

**Figure 14:**
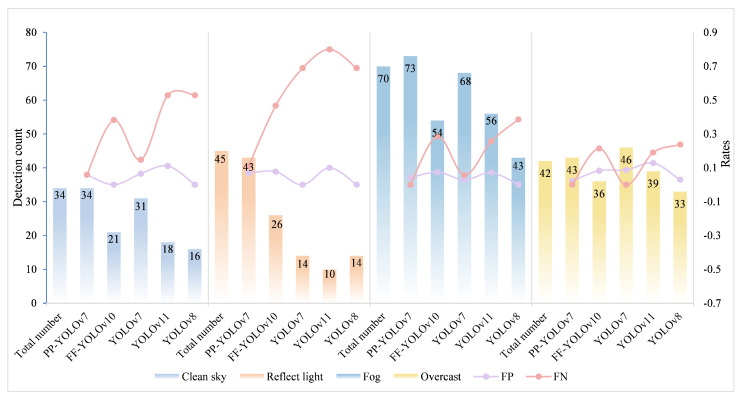
The number of detections, false positive (FP) rate and false negative (FN) rate of each model under different weather conditions.

**Figure 15:**
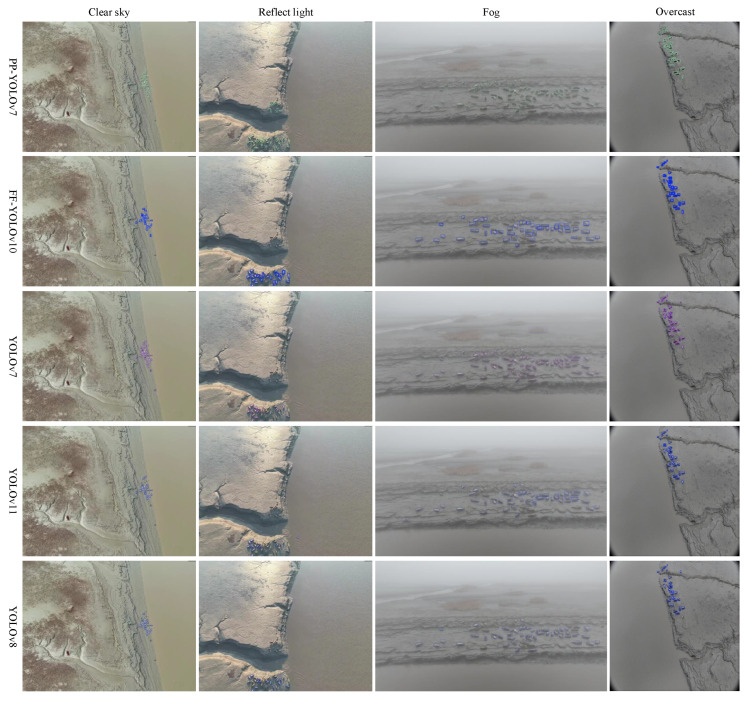
Visualization of detection results of five models under varying weather conditions.

To comprehensively evaluate the robustness and generalizability of the proposed dual-model hierarchical detection framework in real-world marine environments, we conducted tests under four representative weather conditions: sunny, reflective, foggy and overcast. Five mainstream detection models were compared, and performance was assessed in terms of detection completeness, false positives, and missed detections. From the evaluation results presented in Figure 14, it is evident that PP-YOLOv7 consistently demonstrates high detection performance across all weather conditions, particularly excelling in challenging environments. FF-YOLOv10, a lightweight model, delivers faster processing and lower power consumption, making it ideal for real-time detection applications. The visualized detection outcomes under each condition are shown in Figure 15. As shown, our framework demonstrates consistently high detection completeness across varying seal sizes, even with low-contrast or occluded backgrounds. Furthermore, it substantially reduces both false positives and missed detections compared with baseline methods. These results confirm the framework’s strong adaptability to challenging marine conditions, reinforcing its practical applicability for UAV-based ecological monitoring tasks.

## 4. Discussion

This study introduces a dual-model hierarchical detection framework that combines a lightweight model (FF-YOLOv10) with a high-precision model (PP-YOLOv7) to enhance the efficiency and accuracy of spotted seal monitoring in natural environments. The framework effectively addresses key challenges in small-object detection, including feature degradation, the low pixel resolution of spotted seals in UAV imagery, and the computational constraints of edge devices. Experimental results show that FF-YOLOv10 achieves an inference speed of 833.3 FPS while maintaining detection accuracy, and reduces the number of parameters by 24.2% compared with the original YOLOv10. In addition, PP-YOLOv7 increases detection accuracy by 1.2% without adding computational complexity. By balancing real-time responsiveness with detection completeness and accuracy, the proposed framework fulfills the core requirements of UAV-based wildlife monitoring under field conditions.

The framework’s robustness was further validated by testing it under various weather conditions, including sunny, reflective, foggy, and overcast environments. The results demonstrated that the proposed model effectively handles low-contrast backgrounds and varying environmental factors, with FF-YOLOv10 excelling in real-time detection and PP-YOLOv7 ensuring high detection accuracy, even under challenging conditions. These results confirm the framework’s practical applicability and adaptability in diverse field settings, where environmental factors such as lighting, background interference, and weather conditions often vary.

Compared with conventional object detection methods such as YOLOv11 and RT-DETR, FF-YOLOv10 achieves a substantial reduction in model complexity while maintaining detection accuracy. This improvement primarily benefits from the introduction of the C2f module, which reduces redundant channel connections and incorporates cross-layer feature fusion to achieve a more lightweight network structure. Additionally, the Focal Modulation mechanism enhances the model’s ability to represent small objects and complex backgrounds by integrating spatial attention guidance and modality-specific modulation. PP-YOLOv7 further contributes to performance gains by introducing a dedicated small-object detection head and partial convolution modules, markedly improving detection accuracy for spotted seals—targets that are small in scale and often embedded in visually similar backgrounds within UAV imagery. This approach shares conceptual similarities with the feature enhancement strategy proposed by Liu et al. [51], which improves salient region extraction through multi-scale and edge-aware attention in marine species recognition. Such alignment underscores the significance of enhancing localized perceptual sensitivity for detecting small targets under background similarity interference. Similarly, Zhang et al. [52] highlighted that in environments with substantial background interference, the incorporation of a dedicated small-object detection head significantly enhances detection performance.

The dataset used in this study was collected from a relatively limited geographic location and primarily focuses on a single species—the spotted seal. Therefore, the application scope of the proposed approach still holds considerable potential for expansion. Future research could explore validation across diverse geographic regions and species contexts to enhance the generalization and adaptability of the model. The integration of infrared imaging technology into the proposed dual-model hierarchical detection framework warrants further investigation, as it enables continuous monitoring under low-light or nighttime conditions. This approach is expected to preserve detection accuracy while minimizing system energy consumption and data redundancy, thereby advancing the practical deployment of this technology in ecological conservation and biodiversity management.

## 5. Conclusions

This study presents a dual-model hierarchical detection framework for the sustainable, real-time monitoring of spotted seals. By deploying two enhanced object detection models—PP-YOLOv7 on ground-based workstations and FF-YOLOv10 on UAV platforms—the framework enables rapid detection and accurate identification of seals without disrupting their natural behavior. Experimental results show that the lightweight design significantly improves computational efficiency through staged feature allocation and selective computation, resulting in a 14.3% increase in FPS while maintaining stable detection accuracy. This design facilitates real-time onboard inference on UAVs and reduces the impact of data transmission and storage on flight endurance. The precision-focused detection model incorporates multi-scale feature maps and hierarchical convolution operations to capture fine-grained details of small targets. It achieves 94.2% detection accuracy and a 1.9% improvement in recall, all without increasing computational complexity. The model also enhances sensitivity to small objects, especially under conditions of background–target color similarity. In comparison with traditional monitoring approaches, the proposed framework offers superior detection precision, better real-time responsiveness, and higher automation. Future research will focus on further optimizing the lightweight design for UAV models to extend flight endurance and adapt to complex environmental conditions, thereby improving its performance in field-based monitoring applications.

## Figures and Tables

**Table 1:** Experimental configuration environment.

Configuration	Parameter
Programming language	Python 3.8.18
Deep learning frameworks	PyTorch 1.8.0
Operating system	Windows 10 X64
CPU	Intel i9-10980XE
Host memory	64 GB
GPU	Nvidia GeForce RTX 3080Ti

**Table 2:** Comparison of base model detection results. Among them, the YOLO series all use version n.

Model	Precision	Recall	mAP	GFLOPs	MB	Parameters	FPS
YOLOv11	0.857	0.654	0.735	6.3	5.5	2,582,347	666.7
YOLOv10	0.867	0.672	0.741	1.2	5.4	2,492,822	625.0
YOLOv8	0.856	0.668	0.742	8.1	6.3	3,005,843	769.2
YOLOv7	0.930	0.847	0.901	103.2	74.8	36,479,926	166.7
YOLOv5	0.920	0.726	0.810	15.8	14.4	7,012,822	108.1

**Table 3:** Comparative performance of object detection algorithms on the seal dataset.

Model	Recall	mAP	MB	Parameters	FPS
Spike-YOLO	0.609	0.696	27.1	13,248,643	196.1
YOLOv7-tiny	0.688	0.793	12.3	6,006,646	212.8
RT-DETR	0.683	0.753	66.2	31,985,795	81.3
SSD	0.465	0.691	90.6	23,612,246	25.2
**FF-YOLOv10**	0.665	0.742	**5.0**	**1,888,742**	**833.3**

**Table 4:** Ablation experiment results of FF-YOLOv10.

Model	Recall	mAP	MB	Parameters	FPS
YOLOv10	0.672	0.741	5.4	2,492,822	625
F-YOLOv10	0.664	0.748	4.8	1,780,323	212.8
FF-YOLOv10	0.665	0.742	5.0	1,888,742	833.3 ↑33.3%

↑ indicates an increase in FPS.

**Table 5:** Ablation experiment results of the improved YOLOv7 model.

Models	Precision	Recall	mAP	MB	Parameters
YOLOv7	0.93	0.847	0.901	74.8	36,479,926
YOLOv7+A	0.937	0.874	0.928	72.5	37,023,248
**YOLOv7+A+B**	**0.942**	0.866	0.924	**72.5**	**37,023,184**

A represents the small-object detection layer, and B represents partial convolution.

## Data Availability

The data presented in this study are available upon reasonable request from the corresponding author. Due to restrictions imposed by the ecological reserve, only partial datasets can be provided.
